# A light‐touch routing optimization tool (RoOT) for vaccine and medical supply distribution in Mozambique

**DOI:** 10.1111/itor.12867

**Published:** 2020-09-01

**Authors:** Larissa P.G. Petroianu, Zelda B. Zabinsky, Mariam Zameer, Yi Chu, Mamiza M. Muteia, Mauricio G.C. Resende, Aida L. Coelho, Jiarui Wei, Turam Purty, Abel Draiva, Alvaro Lopes

**Affiliations:** ^1^ Department of Industrial & Systems Engineering University of Washington Seattle WA 98195‐2650 USA; ^2^ VillageReach Headquarters, 2900 Eastlake Ave. E, Suite 230 Seattle WA 98102 USA; ^3^ VillageReach Mozambique Office Rua das Rosas, 105 ‐ Sommerschield 2 Maputo Mozambique; ^4^ The Information School University of Washington Seattle WA 98195‐2840 USA

**Keywords:** humanitarian logistics, vehicle routing, vaccine distribution, medical supplies distribution, routing tool, indexing algorithm, route optimization

## Abstract

Planning vaccine distribution in rural and urban poor communities is challenging, due in part to inadequate vehicles, limited cold storage, road availability, and weather conditions. The University of Washington and VillageReach jointly developed and tested a user‐friendly, Excel spreadsheet based optimization tool for routing and scheduling to efficiently distribute vaccines and other medical commodities to health centers across Mozambique. This paper describes the tool and the process used to define the problem and obtain feedback from users during the development. The distribution and routing tool, named route optimization tool (RoOT), uses an indexing algorithm to optimize the routes under constrained resources. Numerical results are presented using five datasets, three realistic and two artificial datasets. RoOT can be used in routine or emergency situations, and may be easily adapted to include other products, regions, or logistic problems.

## 1 Introduction

Distribution of vaccines in rural Mozambique faces many challenges such as inadequate vehicles, limited cold storage, road availability, and variable weather conditions. This paper presents joint work between VillageReach, a nonprofit organization that transforms health care delivery to reach everyone, including the most rural and remote communities (VillageReach, 2019), and the University of Washington (UW), Department of Industrial and Systems Engineering, to optimize delivery routes that can improve the efficiency of vaccine distribution when considering issues such as vehicle availability and reliability, road conditions, and weather. VillageReach and the UW are working with the Mozambican Ministry of Health (MoH) to develop and test a user‐friendly, Excel spreadsheet based optimization tool called the route optimization tool (RoOT), for routing and scheduling to effectively distribute vaccines and other medical commodities to health centers across the country. RoOT is designed to be easily updated and executed, and considers the availability of roads, vehicles, and medical products to distribute. The tool can be used periodically for routine operations, in emergency situations, or pandemics such as COVID‐19 (Peckham, 2020). RoOT can also be used for strategic planning when exploring the effect of changes in the situation (such as new or closed health centers, additional or fewer vehicles, new medical supplies, or new refrigerators) on distribution plans.

## 2 Methodology

The process of creating RoOT started with several discussions among UW and VillageReach team members describing goals for a light‐touch routing tool for potential government users in Mozambique. Based on the experience of VillageReach and the Mozambican government with current network optimization tools, it became clear that the user interface must not be complicated to use and that it should not be difficult to update data. Existing tools are not typically used by the Mozambican MoH to plan distribution of medical supplies, partly because of these difficulties. The team decided to consolidate all input data into one Excel spreadsheet file that is easy for users to update. The spreadsheets are designed to be in a similar format to documents that the government stakeholders are familiar with, to make data entry easy. The output results are presented in another Excel file.

The objectives and constraints of the route optimization model were discussed with VillageReach, UW, and MoH team members, to ensure that the model reflects issues of concern to the end users. Road and vehicle conditions are important considerations and affect delivery plans. Certain roads may not be accessible due to rain or flooding, and a different route may be needed during the rainy season or in the event of cyclones. Furthermore, untimely delivery may affect the potency of vaccines. For example, if a vehicle breaks down en route or gets stuck in the mud, the temperature of the vaccines in cold storage may violate the recommended range, which would impact the potency of the vaccines (Garnett, 2015). These critical and practical issues that affect distribution are included as “risk factors” and are incorporated into the route optimization model with the use of penalty parameters in an objective function.

Another objective is to minimize the total transit time to distribute the vaccines to the rural areas. RoOT allows users to select either objective function, or to minimize a weighted sum of the two objective functions. Although most routing problems minimize cost, the primary objective in RoOT is the timely delivery of effective vaccines within constrained resources. However, a cost calculation associated with a solution is provided to the user as additional information.

The first prototype was shared with all team members over several conference calls, and then the UW doctoral student traveled to Mozambique to obtain more feedback and determine important features. As is typical with vehicle routing optimization models, the computer execution time to determine an optimal solution can be hours or even days, which is impractical for our end users. It was determined from interviews with VillageReach and MoH that a light‐touch tool must *quickly* return a feasible solution so that it can be useful for operational decisions. Instead of using a commercial solver, a new solver, called vehicle routing and scheduling algorithm (VeRSA) (Zabinsky et al., 2020), was tailored to fit the considerations of vaccine distribution. VeRSA uses an indexing method to determine near‐optimal feasible solutions promptly and is embedded into a branch‐and‐bound framework to obtain an optimality gap for intermediate solutions. Given sufficient time, it will eventually obtain an optimal solution (Zabinsky et al., 2020). The indexing algorithm is coded in Python and reads and outputs Excel files. For comparison purposes, the performance of the Python implementation is compared to the performance of a commercial solver, Gurobi 8.0.1 (Gurobi Optimization, 2019), as well as to two open‐source solvers, CBC and GLPK (Forrest et al., 2018; GNU, 2019), on the same mixed‐integer optimization model.

RoOT was tested by VillageReach team members during the summer of 2019, and feedback was incorporated into the version delivered on 1 November 2019. Users from the Mozambican MoH are being trained on the use of RoOT and final modifications will be implemented in 2020. RoOT will be translated into Portuguese for the Mozambican users, and the English version will be available on Github in 2020 for other users from NGOs, government, and academia.

This paper is organized as follows. Section 3 contains background material and a brief literature review of humanitarian logistics with a brief discussion of vehicle routing problems (VRPs) and algorithms. Section 4 includes a detailed description of RoOT. The mixed‐integer optimization model is given in Section 5, and the indexing algorithm is discussed in Section 6. Numerical results comparing the performance of several solvers using three realistic datasets are presented in Section 7, and finally, conclusions are drawn in Section 8, followed by future work in Section 9.

## 3 Background and literature review

### 3.1 Humanitarian logistics

Vaccine distribution is a difficult problem for governments around the world, but it is especially challenging in poor neighborhoods and low‐ and middle‐income countries, where the demand is uncertain due to lack of accurate population estimates, and road infrastructure is poor, even inaccessible under some weather conditions. Chan et al. (2013) discuss the problem that low‐ and middle‐income countries have of adopting new vaccines. For example, 98% of newborns in low‐income countries do not receive pneumococcal conjugate vaccines, according to their government plan, while in high‐income countries, the number is 13%. The geography of many low‐income countries, such as the lack of proper roads or transportation methods to reach populations in need (Chan et al., 2013), is an important factor. In addition, current distribution respects political boundaries (Lim et al., 2019). Therefore, it is important to understand this type of humanitarian logistics problem and adapt how other fields achieve efficient distribution under these conditions.

Even though it is important to understand the underlying conditions in this type of humanitarian logistics problem, it is also vital to recognize the available supply‐chain tools that can improve operations in these circumstances. Van Wassenhove (2006) and Tomasini and Van Wassenhove (2009) discuss the existing gap between supply‐chain tools for humanitarian organizations and those used in the private sector. Humanitarian organizations have begun to realize the value of logistics and supply‐chain management tools used in the private sector to improve their operations (Van Wassenhove and Pedraza Martinez, 2012). Consequently, humanitarian organizations have begun to adopt private sector practices in their operations.

For example, using supply‐chain practices from the private sector, Nigeria has increased its immunization coverage by about 30%, with a cost reduction of about 15% (Sarley et al., 2017). Using computer simulation, Lee et al. (2016) redesigned the vaccine distribution process in two provinces in Mozambique, as a joint effort with VillageReach. The redesign increased availability by 27% and 8%, while decreasing costs by 40% and 37%, respectively. However, some differences between a private sector supply chain and a humanitarian relief chain should be noted. In the private sector, the network configuration is more stable with respect to supply and demand (quantities and entities involved), while they are challenging to predict and are less consistent in humanitarian logistics (Manopiniwes and Irohara, 2014). In addition, cost is often the sole objective in the private sector, whereas a humanitarian relief chain may prioritize rapid distribution using available resources (Tomasini and Van Wassenhove, 2009).

Limited technology is an important consideration for humanitarian logistics, such as poor Internet connectivity, lack of real‐time data, and outdated computers. Supply‐chain tools need to be simple for users, and may not be adopted by the end users if they are complex to use, require data that are not easily available, require time to update, or need a fast reliable Internet connection. There is a lack of tools for routing that are easy to use and address desired issues that were highlighted during interviews with VillageReach staff and Mozambican MoH officials (Vitoriano et al., 2013).

**Fig. 1:**
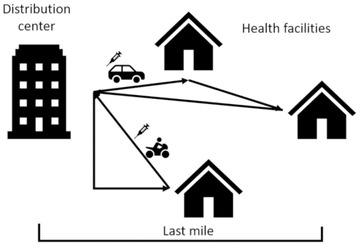
Last mile of vaccine delivery.

The differences between humanitarian organizations and private sector, and the lack of available tools motivate the development of an easy‐to‐use routing tool that is designed to address last mile (Laseinde and Mpofu, 2017) vaccine distribution in a developing country. Figure 1 exemplifies the last mile for vaccine delivery.

### 3.2 Current tools

We evaluated 33 commercial software products listed in Tables 1 and 2, where Table 1 lists 15 software products that focus on humanitarian logistics and Table 2 lists 18 software products that focus on routing. Most of the software that provide decision support systems for humanitarian logistics focus on inventory control and are primarily used for disaster preparedness and response but do not incorporate routing. These systems also primarily address management but not operational issues (Vitoriano et al., 2013). Therefore, this project focused on developing a routing optimization tool that is simple to use, and in which the user interacts only with Excel files. The optimization algorithm is run in Python in the background to create the distribution routes and schedules.

**Table 1:** Humanitarian logistics tools/software

	Tool/software name
1.	LLamasoft—supply‐chain Guru—cloud‐based supply‐chain design software
2	HERMES—highly extensible resource for modeling event‐driven supply chains
3.	GLC—global logistic competence
4.	SUMA
5.	LSS
6.	Fritz Institute—Humanitarian Logistics Software (PRSRM‐HLS)
7.	HELIOS
8.	Sahana
9.	Chevinfleet
10.	Logistimo
11.	Parcel Project
12.	UNICEF
13.	ELIST
14.	DMIS
15.	LOGITIX

**Table 2:** Vehicle routing tools/software

	Tool/software name
1.	ClearD Optima
2.	DISC
3.	Intelligent routing
4.	JOpt
5.	ODL Studio
6.	OptimoRoute
7.	Optrak4
8.	Routist
9.	Routyn
10.	Scientific logistics cloud‐based route optimization
11.	StreetSync Pro
12.	Locus Dispatcher
13.	Workwave Route Manager
14.	Onfleet
15.	Routific
16.	Loginext
17.	Track POD
18.	Cro software solutions

### 3.3 Vehicle routing problem

The problem addressed in this project is a VRP in which routes between centers are planned such that each center is visited once by a vehicle, and each vehicle starts at the distribution center (often termed as a depot) and returns to it by the end of its route. This VRP is widely studied and Laporte (1992) presents a comprehensive review of the problem. There are many variations of the VRP that are studied, such as the addition of time constraints including time windows for delivery, total time for each route (Solomon and Desrosiers, 1988; Kohl and Madsen, 1997), and capacity constraints (Laporte et al., 2002; Sungur et al., 2008).

The problem considered in this paper includes capacity constraints on the vehicles and can be formulated as a capacitated VRP (CVRP) (Laporte et al., 2002). The need of a cold chain is also an important aspect to consider in vaccine distribution (Lim et al., 2019). Vaccines are perishable and inappropriate refrigeration outside of ideal storage temperatures results in waste (Comes et al., 2018). Since the vehicles in Mozambique use “cold boxes” as passive containers to keep the vaccines within the proper temperature range, we consider the capacity of cold storage by vehicle type. The size of the vehicle (e.g., motorcycle, car, truck) determines the limited total capacity for its passive container for cold storage and other medical supplies that do not require refrigeration. Therefore, we incorporate two types of capacity constraints per vehicle, called cold and dry capacities. Prosser et al. (2017) discuss the importance of redesigning vaccine supply chain in Benin since insufficient cold chain capacity jeopardized the distribution of new vaccines.

Large and small cold boxes can maintain the proper temperature for a specified maximum amount of time before the vaccines are either used or transferred to a refrigerator at a health center. In this model, the total time of a route is constrained so that the cold box will preserve the vaccine until final delivery. We do not allow transfer of products between health centers, as it would require intermediary storage and some regions do not have electricity. Therefore, in addition to the vehicle capacity constraint, we also include timing constraints, such as a constraint on the time from departure to the time of delivery and a constraint on the time for a driver to complete a route (typically eight hours) as in Laporte (1992), Laporte et al. (1992), Bräysy and Gendreau (2005), Chen et al. (2006), Grasas et al. (2014), and Zabinsky et al. (2020).

Different factors can lead to vial wastage in vaccine distribution. In addition to refrigeration or cold boxes keeping the vaccines within a proper temperature range, closed vial wastage may be due to breakage of vials (Hanson et al., 2017). We include the risk of breakage of vials due to poor road condition or poor vehicle condition with a penalty parameter.

In humanitarian logistics, most routing problems focus on disaster preparedness, for example, earthquakes (Mete and Zabinsky, 2010; Ahmadi et al., 2015; Tofighi et al., 2015). These problems are commonly modeled as classical vehicle routing or dynamic network problems, having as objective the minimization of total travel time, unmet demand, or cost (Ozdamar and Ertem, 2015). Hoyos et al. (2015) consider the use of operations research in disaster operations management. In 48 papers using mathematical models, the most common goals were to minimize cost (31%), minimize unmet demand (21%), and maximize regional coverage (19%).

The model presented in this paper has two objective functions, that is, minimization of transportation time and minimization of risk factor for spoilage and breakage of vaccine using penalties for use of certain roads and vehicles. Although much has been written about the importance of transportation for humanitarian logistics, infrastructure risks such as information technology, financial systems, and transportation are rarely addressed, and those risks are responsible for most of the network disruptions. Since transportation is fundamental to humanitarian logistics, its risks should be properly accounted for (Baharmand et al., 2017). According to Ozdamar and Ertem (2015), road risks should be considered in the objective function, along with cost, travel time, and demand satisfaction. Furthermore, road failure, caused by flooding, road sink, or bridge collapse, could make a calculated route longer than expected or even infeasible (Hamedi et al., 2012). An option is to consider the reliability of the transportation scheme, such as the probability of not completing a route. Penalty parameters are also used to incorporate a failure probability as in Hamedi et al. (2012). The use of penalty parameters reduces the amount of traffic (e.g., number of vehicles) on unreliable roads (Hamedi et al., 2012). Another way to calculate risk is to estimate the probability a road between two centers is inaccessible. Nolz et al. (2011) identify critical roads that could be bottlenecks in a tour. Avoiding the bottleneck decreases the total risk. In one example, using a minimum risk approach, the risk range decreased from 0.97–0.98 to 0.81–0.83. However, the travel time range increased from 0.25–0.61 to 0.59–0.98. Since there is a trade‐off between risk and travel time, the users of RoOT will decide whether to minimize risk (e.g., penalties) or minimize transit time, or minimize a weighted sum of both objectives.

While analyzing risks for humanitarian logistics is not typically addressed in the literature (Baharmand et al., 2017), risk minimization is one of the main goals when transporting hazardous materials. Since the 1970s, the National Transportation Safety Board recommends a risk‐based approach for transporting hazardous materials (List et al., 1991). One of the reasons there is a large quantity of research done for risk minimization is that fatalities due to hazmat‐related traffic accidents are considered unacceptable (Akgün et al., 2007). Among multiple objectives for routing hazardous materials, minimization of risk should be the main objective (Patel and Horowitz, 1994).

Some approaches for risk analysis for hazardous material transportation incorporate an evaluation of accident rates by mode, carrier type, vehicle type, and road classification (List et al., 1991). According to List et al. (1991), private vehicles have lower accident rates than for‐hire vehicles, and accident rates due to the time of day and weather conditions depend on the roadway type. In addition, accident rates may consider the road classification (expressways, arterials, collectors, ramps), the designed speed, the surface condition, and visibility (Saccomanno and Chan, 1985).

Considering the weather and time of day (e.g., daylight or night) is also important. Weather affects not only the transit time but also the risk of an accident. This includes the risk of the harm that a hazardous spill can do to the nearby population (Akgün et al., 2007). Moreover, other risks that may be addressed are the probability of an accident or delay at a facility, the accident rate en route, and the probability of an accident due to speed and road condition (Batta and Chiu, 1988).

Therefore, to minimize the spoilage and breakage of vaccines, the methodology used for hazardous materials was applied and vehicles and road were classified according to their conditions, assigning corresponding penalties to each. The corresponding objective function representing risk is the minimization of the sum of these penalties.

### 3.4 Exact methods and heuristics for solving vehicle routing problems

The VRP is in general NP‐hard and is difficult to solve exactly for instances with more than 50 customers (Laporte et al., 2002). Exact methods that guarantee an optimal solution usually start with a relaxation of the linear problem, followed by a presolve phase to reduce the problem. Then they apply branch‐and‐bound, branch‐and‐cut, or cutting‐plane algorithms to solve the problem exactly (Martin, 2010; Forrest et al., 2018; GNU, 2019; Gurobi, 2019). Research on solving large‐scale VRPs often focuses on heuristic solution approaches, which do not guarantee optimality but can find good solutions quickly to large‐scale problems. Cordeau et al. (2002) and Vidal et al. (2013) present extensive comparisons of heuristics applied to solve VRPs. Common heuristics are tabu search, genetic algorithms, and greedy randomized adaptive search procedure (GRASP) (Kontoravdis and Bard, 1995; Taillard et al., 1997; Gendreau et al., 1999; Berbeglia et al., 2010; Vidal et al., 2012; Grasas et al., 2014; Hanson et al., 2017).

To address large‐scale problems, this paper applies a variant of the VeRSA presented in Zabinsky et al. (2020). VeRSA is an exact method that embeds an indexing rule to prioritize pickups on different routes in a branch‐and‐bound framework, dynamically constructing the branches to be traversed. Therefore, it is possible to reach a near‐optimal feasible solution quickly, while guaranteeing an optimal solution if the user runs it long enough. In Zabinsky et al. (2020), the performance of VeRSA compared favorably to a commercial solver and a genetic algorithm. In this paper, we adapted the indexing algorithm used in VeRSA to our vaccine distribution and routing problem, as discussed in Section 6.

## 4 Description of the route optimization tool

### 4.1 Model approach

The routing optimization model in RoOT is a mixed integer program (MIP) with two objectives and constraints tailored to the vaccine distribution problem in Mozambique. The participation of all team members in the modeling effort aided in identifying important considerations. Discussions of how to incorporate data from existing sources into Excel spreadsheets were also critical for the light‐touch tool to be accepted and used.

In every province in Mozambique, vaccine distribution is done by district to respect political boundaries. Three datasets are included in this paper, with one district in each of three provinces (Tete, Maputo, and Sofala). For example, one district in the Sofala province has 16 health centers, 5 vehicles, and 13 products.

From discussions with the stakeholders, it was decided that the model should consider the following:

- *Multiple objectives with different weights*. The users agreed that two objectives are important. The first is to minimize total transit time and the second is to minimize the penalties for using vehicles or roads that are not in good condition. The users can decide how to combine the two objective functions by choosing an appropriate set of weights. The penalty parameter was also discussed. Penalty parameters are used to represent unreliable vehicles and poor road conditions that can jeopardize the delivery of viable vaccines (breakage and temperature range). Since it is difficult for users to scale penalty parameters appropriately, the Excel input file has drop‐down menus and the users select the appropriate vehicle and road condition (e.g., there are four options for a vehicle ranging from very reliable to unreliable). The penalty parameter values are determined in the Python code to maintain appropriate scaling.
- *Supply and demand issues*. It was discussed that the demand projections can exceed the supply, and also that demand can exceed the storage capacity at a health center. Usually, vaccines are distributed once a month in Mozambique, so it was agreed that if there is insufficient supply to meet all of the monthly demand, then the input demand will be scaled back until it reaches the available supply. In this case, the recommendation is to do more than one delivery during the month, when the products arrive. Sometimes the demand at a health center exceeds the current storage capacity at the center, however, it is anticipated that there will be sufficient storage when needed (perhaps a refrigerator is awaiting repair). In this case, a warning is issued but the optimization can still be run.
- *Allow multiple routes per vehicle*. The user inputs all available vehicles, and each vehicle is assigned a route. In addition, each route has a maximum duration time (typically eight hours, specified by the user). If needed to achieve the complete distribution, vehicles can be assigned more than one route, however, all available vehicles should be used before one is reused. For example, if two vehicles are available, with available drivers, each will be assigned a route that may be completed on the same day. However, if the two vehicles do not have enough carrying capacity or time to deliver all of the vaccines, they can be assigned an additional route to complete on another day.
- *Cost*. Although the users do not want to necessarily minimize cost in the objective function, they are still interested in the cost of the routes and distribution plan. Cost calculations are provided in the output file based on input cost parameters. It should be noted that the costs are calculated after defining the routes, and are not part of the optimization model.

### 4.2 Usability

It is important that the tool should be easy to use, and usability of the RoOT led to the decision to use Excel spreadsheets for inputs and outputs. The optimization algorithm is run by clicking on executable Python code, which allows a user to browse and select an input file. An Excel output file is created containing the routes with details on the types and quantities of vaccines and medical supplies to be delivered.

VillageReach evaluated the first prototype according to usability, using methods from Nielsen and Mack (1994). As a result, it was determined that there were too many possibilities for typos by the users, so drop‐down menus were incorporated in the input file for many parameters. Adding a new health center necessitates changes in several of the spreadsheets, so the input file was designed so that a user only enters the name of the new health center once, and it is automatically replicated to the other sheets to avoid errors. Similarly, new vaccines, medical supplies, or vehicles are input only once and then automatically replicated to sheets with connecting cells. Worksheets and cells with calculations that are used by the Python program but are not important to the user are locked and hidden. Sections 3.4 and 3.5 present the input and output files.

### 4.3 Input file

The input file is an Excel file with seven spreadsheets. Each sheet has brief instructions in the first line. The headings that are highlighted in yellow require input from the user, and some of the input cells have drop‐down menus. The seven input sheets are named parameters, products, center_capacities, demand, vehicle, distance_data, and road_condition.

**Fig. 2:**
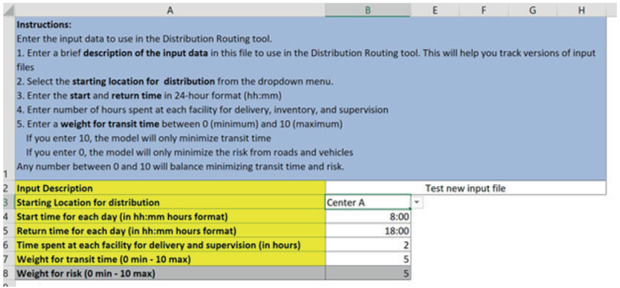
Parameters sheet—input.

The parameters sheet (Fig. 2) defines the general parameters of the VRP, including run description, start and final location, start time and return time for the route, drop‐off time, and weights for each objective function. The weights are used to balance the objective of minimizing transit time with the objective of minimizing penalties for using roads or vehicles that can be risky to the product due to their condition. Any number between 0 and 10 will balance minimizing transit time and risk. The total is always 10. For example, the user can input 6 for the transit time weight, and the sheet automatically calculates 4 for the weight on the penalties. If the user enters 10, the model will only minimize transit time. If the user enters 0, the model will only minimize the penalties for roads and vehicles (risk to vaccines).

**Fig. 3:**
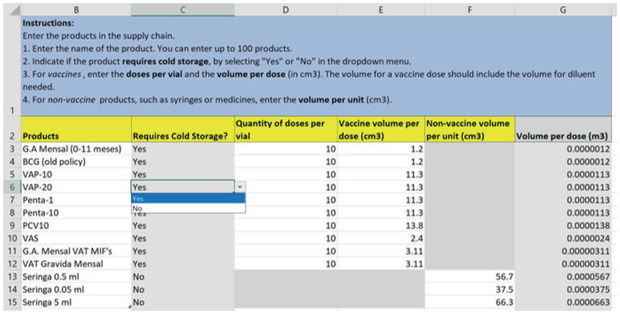
Product sheet—input.

In the products sheet (Fig. 3), the user enters the products to be distributed and their volume and storage characteristics, such as doses per vial or number of syringes. The user also specifies if the product needs cold storage.

**Fig. 4:**
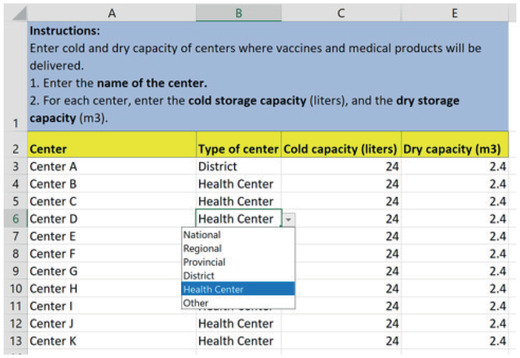
center_capacities sheet—input.

The center_capacities sheet (Fig. 4) has health center information (name and type), and storage capacities for cold and dry products. When a user enters a new center name on this sheet, it is automatically added to the other sheets.

**Fig. 5:**
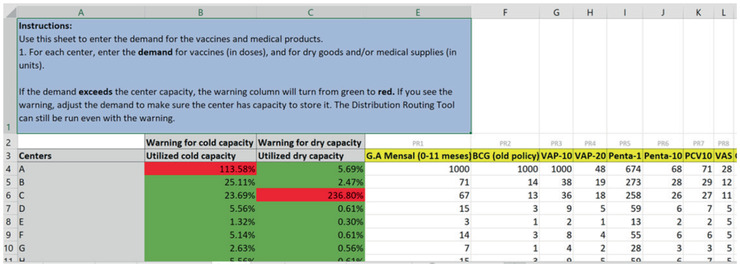
Demand sheet—input.

In the demand sheet (Fig. 5), the user enters the demand for each product to be distributed to each center, in doses or units. If the demand exceeds the center capacity, the warning column will turn from green to red. If there is a warning, the user should adjust the demand or increase the storage capacity to make sure the center can store the delivered vaccines and supplies. Note that the optimization can still be run even if there is a warning.

**Fig. 6:**
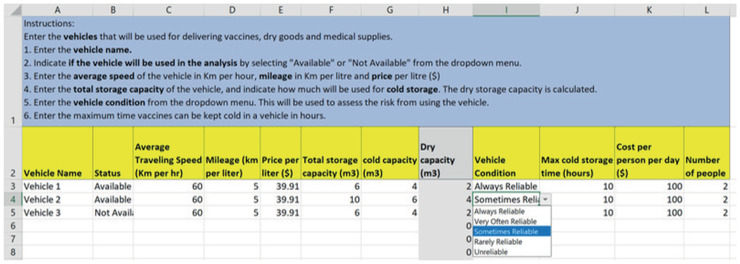
Vehicle sheet—input.

The vehicle sheet (Fig. 6) has the vehicle information that is used for delivering vaccines, their availability, and their characteristics, such as average velocity, fuel consumption, fuel costs, storage capacity, and personnel per diem costs for distribution.

**Fig. 7:**
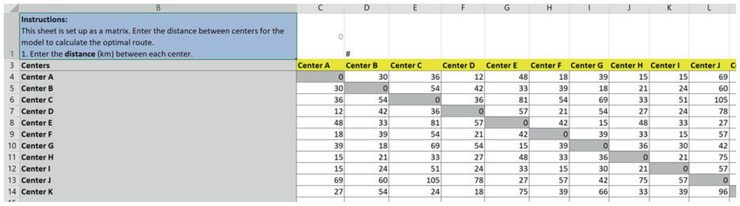
distance_data sheet—input.

The distance_data sheet (Fig. 7) displays the distance between centers as a matrix. To provide flexibility in representing one‐way roads, the distance matrix does not need to be symmetric.

**Fig. 8:**
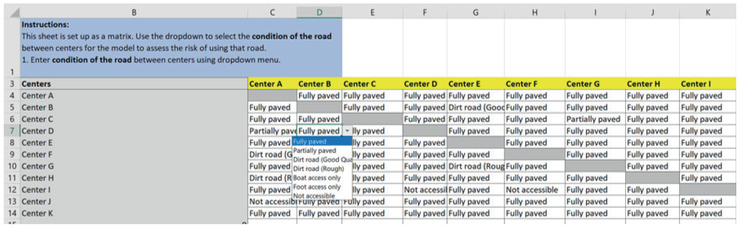
road_condition sheet—input.

Finally, the road_condition sheet (Fig. 8) defines the condition of the road between centers using a drop‐down menu, for the model to assess the risk of using that road. Options include fully paved, partially paved, dirt road (good quality), dirt road (rough quality), boat access only, foot access only, and not accessible.

### 4.4 Output file

**Fig. 9:**
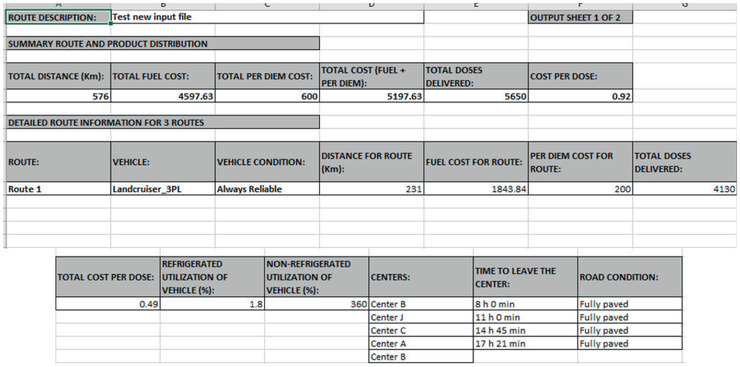
Routes sheet—output.

The Excel output file has two sheets: routes and products to be delivered. The routes sheet, in Fig. 9, gives the recommended routes, including the distances traveled, fuel and personnel costs, utilized vehicle and its condition, utilized capacity per vehicle, dry and cold capacities, and the centers visited, giving the time to leave each of the centers and the road condition between them. In Fig. 9, three routes are recommended. The summary description for all three routes is shown at the top, and the details for the first route are also shown.

**Fig. 10:**
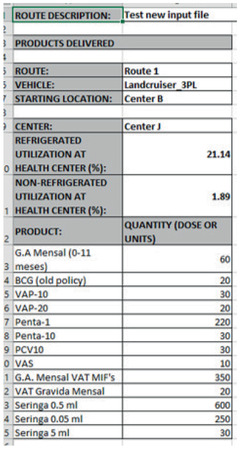
Products sheet—output.

The products sheet gives the quantity of each product distributed to each center in each route, by dose or unit. It also provides the utilized capacity, dry and cold, by center. In Fig. 10, the information of the route is given at the top and the details of the products delivered at the first center on the route, Center J, and its utilized capacity are also shown.

### 4.5 Use cases

While developing the tool, VillageReach team members shared questions or use cases that the Mozambican government users often asked. RoOT was designed so that these questions can be easily answered. The questions include:

- What if my main distribution center changes location?
- What if a new vaccine is added for distribution?
- What if a new facility is added to my current list?
- What if one of my vehicles breaks down?
- What if I add a new vehicle to my fleet?
- What if the cold storage capacity at a health center is reduced?
- What if new refrigerators arrive?
- What if there is an outbreak and a need for immediate distribution?
- What if a road is unavailable?

The user guide explains how to address each of these use cases. The need to easily add a health center, a new vehicle, a new product, or change capacities was instrumental in designing the input sheets. The Excel input file allows the addition in one place that is replicated across sheets.

When the user updates the input file to answer one of these questions by changing vehicle, center, product, or road information, the newly created input file should be saved with a new descriptive name representing the new analysis. Also, the “run description” in the parameters input sheet may be used to describe the changes in parameters, or the question to be addressed. The run description is repeated in the output file to aid in linking input and output files. The output filename has the same name as the input file with “_result_YYYY‐MM‐DD.xlsx” appended. This can assist in keeping track of changes during analysis.

## 5 Mathematical model

The mathematical optimization model defining the problem is described in this section. Table 3 presents the sets, decision variables, and parameters of the model. It is based on the preliminary work presented in Petroianu et al. (2019).

**Table 3:** Model notation—sets, parameters, and variables

Sets	
Health centers—C	i∈C, o is the supply node
Vehicles—V	v∈V
Refrigerated products—$P_{r}$	$p\in P_{r}$
Nonrefrigerated (dry) products—$P_{d}$	$p\in P_{d}$
Products—P	p∈P
Decision variables	
$y_{ijv}$	Binary variable: equals 1 if products are transported
	from i to j using vehicle v; and equals 0 otherwise
$x_{ijvp}$	Quantity of product p transported from i to j using vehicle v
$t_{iv}$	Time that vehicle v leaves health center i
Parameters	
$W_{t}$	Weight for minimizing the total transit time, in [0,1] interval
$W_{p}$	Weight for minimizing the total penalties, $W_{p}=1-W_{t}$
$\mu_{h}$	Average of all transit times, $\mu_{h}=\sum_{v\in V}\sum_{i,j\in C}2h_{ijv}/{\left|V|\;|C\right|}^{2}$
$\mu_{\beta }$	Average of all vehicle penalties, $\mu_{\beta }=\sum_{v\in B}\beta_{v}/\left|V\right|$
$\mu_{\gamma }$	Average of all road penalties, $\mu_{\gamma }=\sum_{i,j\in C}\gamma_{ij}/{\left|C\right|}^{2}$
$d_{ip}$	Demand at health center i for product p
$h_{ijv}$	Average transit time between i and j using vehicle v
$c_{v}^{r}$	Transportation capacity of vehicle v carrying cold products r
$c_{v}^{d}$	Transportation capacity of vehicle v carrying dry products d
l	Maximum time for a route
$k_{p}$	Volume of product p
$a_{ij}$	Route availability: equals 1 if route (i,j) is available;
	and equals 0 otherwise
$\gamma_{ij}$	Penalty for driving between i and j
$\beta_{v}$	Penalty for driving with vehicle v
W	Time for product drop‐off
M	Big number

In preliminary computational experiments, the number of products adversely affected the computation time. To speed up computation, products are classified as needing refrigeration (cold) or not needing refrigeration (dry) in a pre‐processing phase. By grouping products into only two categories, the number of variables in the model is reduced and so is the computation time. This preprocessing, and similar postprocessing is invisible to the user. The inputs can allow any number of products, and the outputs describe the products delivered along each route:

(1) $$\min\;W_{t}\sum_{i\in C}\sum_{j\in C}\sum_{v\in V}e^{-h_{ijv}/\mu_{h}}y_{ijv}+W_{p}\sum_{i\in C}\sum_{j\in C}\sum_{v\in V}\left(e^{-\gamma_{ij}/\mu_{\gamma }}+e^{-\beta_{v}/\mu_{\beta }}\right)y_{ijv}$$

subject to

(2) $$\sum_{i\in C}\sum_{v\in V}x_{ijvp}-x_{jivp}=d_{jp}\;\forall j\in C,p\in P$$

(3) $$\sum_{j\in C\setminus \{o\}}\sum_{p\in P_{r}}x_{ojvp}k_{p}\leq c_{v}^{r}\;\forall v\in V$$

(4) $$\sum_{j\in C\setminus \{o\}}\sum_{p\in P_{d}}x_{ojvp}k_{p}\leq c_{v}^{d}\;\forall v\in V$$

(5) $$t_{jv}-t_{iv}+M\left(1-y_{ijv}\right)\geq h_{ijv}+W\;\forall i\in C\setminus \left\{j\right\},j\in C\setminus \left\{o\right\},v\in V$$

(6) $$t_{iv}+W\left(1-y_{iov}\right)+y_{ijv}\left(h_{ijv}+h_{jov}\right)\leq l\;\forall i\in C,j\in C,v\in V$$

(7) $$\sum_{i\in C}\sum_{j\in C}y_{ijv}-My_{ojv}\leq 0\;\forall v\in V$$

(8) $$\sum_{i\in C}\sum_{j\in C}x_{ijvp}-Mx_{ojvp}\leq 0\;\forall v\in V,p\in P$$

(9) $$\sum_{i\in C}y_{ijv}-y_{jiv}=0\;\forall j\in C,v\in V$$

(10) $$y_{iiv}=0\;\forall i\in C,v\in V$$

(11) $$y_{ijv}\leq a_{ij}\;\forall i\in C\setminus \left\{j\right\},j\in C,v\in V$$

(12) $$My_{ijv}-x_{ijvp}\geq 0\;\forall i\in C,j\in C,v\in V,p\in P$$

(13) $$y_{ijv}\in \left\{0,1\right\}\;\forall i\in C,j\in C,v\in V$$

(14) $$x_{ijvp}\geq 0\;\forall i\in C,j\in C,v\in V,p\in P$$

(15) $$t_{iv}\geq 0\;\forall i\in C,v\in V.$$

The objective function (1) is a weighted sum of total transit time and total sum of all penalties for chosen vehicles and roads during transit. In the objective function, the exponential and the division by the means are used to normalize the parameter values and consider them in the same scale. Constraints in (2) guarantee that the center demand at each center is met. Constraints in (3) and (4) limit the amount or quantity that vehicle v can carry of cold and dry products, respectively. Constraints in (5) give the time sequence between two sequential health centers. This means that if health center j follows i, the time that vehicle v leaves j has to be greater than the time that it passed by i plus the transit time between the centers and the time for product drop‐off. Constraints in (6) guarantee that the vehicle will always have time to return to the supply node o, while respecting the maximum time for the route l. Constraints in (7) and (8) ensure that each vehicle must depart from the main health center o. In these constraints, M is a large number. Constraints in (9) require that if a vehicle enters a health center, it has to leave it, and constraints in (10) forbid a vehicle from returning to a center immediately after leaving it. Constraints in (11) limit the routes to the available routes, and constraints in (12) ensure that a vehicle v traverses arc (i,j) whenever products are carried between them. Constraints in (13)–(15) define binary variables $y_{ijv}$ and nonnegative variables $x_{ijvp}$ and $t_{iv}$.

## 6 Indexing method

The indexing algorithm used in RoOT is a variation of VeRSA, presented in Zabinsky et al. (2019, 2020). A detailed description of VeRSA and the variation used in this work can be found in Petroianu (2020). To summarize, VeRSA uses an indexing method to quickly construct a feasible solution. By incorporating aspects of the objective function into the index, the feasible solution typically has good performance.

The indexing method created for this problem is based on the mathematical model objective functions and constraints. It is divided into two stages. The first stage defines which vehicle will be used. The index value for choosing a vehicle is calculated according to

(16) $$e^{-\beta_{v}/\mu_{\beta }}+e^{c_{v}^{r}/\mu_{c^{r}}}+e^{c_{v}^{d}/\mu_{c^{d}}}+e^{v_{v}/\mu_{v}},$$

where $\beta_{v}$ is the penalty for vehicle v, $c_{v}^{r}$ is the capacity of vehicle v for refrigerated vaccines, $c_{v}^{d}$ is the capacity of vehicle v for dry goods, and $\mu_{\beta },\mu_{c^{r}},\mu_{c^{d}}$, and $\mu_{v}$ are the averages, respectively, to scale appropriately. The vehicle with the largest index value is assigned a route next. The index prioritizes vehicles with lower penalties and higher capacities and velocity. When all available vehicles are assigned a route, in order of the index, then they may be assigned a second route.

After deciding the vehicle, its route is created using the index calculated in

(17) $$W_{t}e^{-h_{ijv}/\mu_{h}}+W_{p}e^{-\gamma_{ij}/\mu_{\gamma }},$$

where i is the current center, j is the next center to visit, v is vehicle, $h_{ijv}$ is the transit time from i to j using vehicle v, $\gamma_{ij}$ is the penalty for road (i,j), $\beta_{v}$ is the vehicle penalty, and $W_{t}$ and $W_{p}$ are the weights for transit time and penalty objective functions, respectively. The averages, $\mu_{h},\mu_{\gamma }$, and $\mu_{\beta }$ are used to scale appropriately. The node with the highest index value is added to the route. This index prioritizes closer centers and those roads and vehicles with low penalty values.

The choices of vehicle and next node to add to the route have to respect vehicle capacities, time limits, and road availability. These constraints are considered in a feasibility check that is performed every time a new node or vehicle is added to the route. The indexing algorithm constructs a feasible solution by assigning routes until the delivery of products is complete.

The indexing algorithm allows multiple routes per vehicle, but it uses all available vehicles before reusing any of them. This constraint does not appear in the mathematical model (1)–(15). However, in the numerical results, the vehicles were similar, and due to the sizes of the problems, each vehicle was used at most one time. Therefore, the results are comparable.

**Fig. 11:**
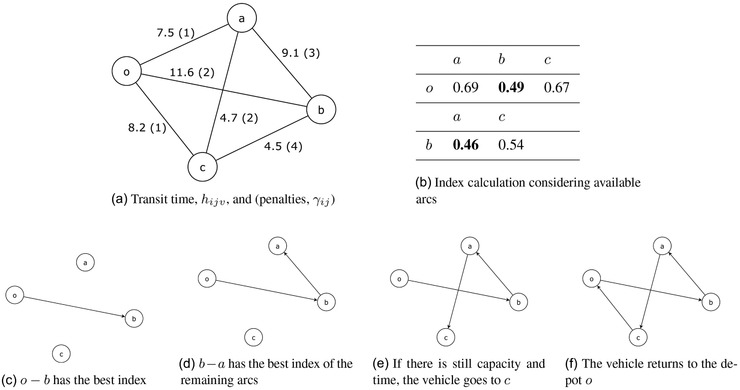
Example of how to construct a feasible solution using the index.

To illustrate how to construct a feasible solution using the index, consider an example with four health centers (*o, a, b, c*), where o is the supply node (i.e., depot). Figure 11a gives the transit time, $h_{ijv}$, and the penalties, $\gamma_{ij}$, between centers. In this example, there is one vehicle with penalty, $\beta_{v}$, equal to 1.

In Fig. 11b, the index values calculated using (17) leaving node o and going to *a, b, c* are shown in the first row. The largest value, 0.49, indicates that node b is visited next, as illustrated in Fig. 11c. Then the index from b to *a, c* is calculated, and the largest value of 0.46 indicates that node a is added to the route. Every time that a center is added, the index is calculated for all remaining centers, and the feasible center with highest index is added to the route, as shown in Fig. 11.

In the indexing algorithm presented in this paper, the algorithm traverses the tree by calculating an incumbent solution for each initial branch of the tree, a depth evaluation of the branch. Then it creates an elite set with 12% of the incumbent solutions. This elite set is divided into two blocks of solutions. From its total, 50% come from the best incumbent solutions and 50% are solutions considering the largest solution uncertainty intervals. The uncertainty interval is the local best solution minus local lower bound. Each solution of this elite set represents a complete branch that will be explored in search of better solution and will help to traverse the tree. The global lower bound of the problem is defined as a minimum spanning tree in which the cost is related to the index in (17). The local lower bound is calculated exploring the current branch up to the its current level.

This indexing algorithm presents good results in comparison to the solvers for the same mathematical model, finding the optimal solution promptly for small datasets, and obtaining better optimality gaps for larger sets, as is demonstrated in Section 7.

## 7 Numerical results

In this section, three realistic datasets with information from Mozambican provinces were used as inputs to perform computational experiments. District A has 11 health centers, 13 products, and 1 vehicle. District B has 16 health centers, 13 products, and 2 vehicles. District C has 13 health centers, 12 products, and 6 vehicles, as shown in Table 4. The three datasets have the same penalties for all vehicles and roads. As mentioned in Section 4, the products are grouped into two types: refrigerated and nonrefrigerated. Therefore, the models consider only two types of products while optimizing the routes. It is valid for all datasets.

**Table 4:** Size of test datasets

Dataset	Number of centers	Number of vehicles	Number of variables	Number of constraints
District A‐small	8	1	200	1165
District B‐small	8	2	400	2330
District C‐small	8	3	600	3495
District A	11	1	374	2260
District B	16	2	1568	9766
District C	13	6	3120	19,140
50 centers simple	50	5	24,200	157,025
50 centers modified	50	5	24,200	157,025

The computational experiments compared the runtime and solution found by solving the same MIP by Gurobi 8.0.1, CBC, GLPK, and RoOT. All the tests were run on a Dell XPS13 computer, Intel CORE i7, with 16 GB of RAM. The weighted objective function used $W_{t}=W_{p}=0.5$ for all tests.

Due to the problem sizes, optimality can be found in less than four hours only for District A. Therefore, three smaller datasets were created to check if the indexing algorithm could discover an optimal solution that was confirmed using Gurobi. The smaller datasets are based on a subset of centers in each district, called District A‐small, District B‐small, and District C‐small. The size of each dataset is given in Table 4.

Two large test datasets with 50 health centers and 5 vehicles were also created to compare the solvers on large instances. The first large instance, called 50 centers simple, has identical vehicles and the penalties for all the roads are same. The second instance, called 50 centers modified, has five different vehicles and different penalties for the roads. All the instances are available online, see Petroianu (2019a).

For the small datasets, Gurobi was able to solve the MIP to optimality, as shown in Table 5. The indexing algorithm in RoOT found the same optimal solutions quickly for the three small datasets, but did not confirm optimality within 30 minutes of runtime. The open‐source solver CBC also discovered the same optimal solutions, although taking more time, and did not confirm optimality within 30 minutes of runtime. The other open‐source solver GLPK was able to discover the same optimal solutions for two of the three datasets, but reported an infeasible solution as “optimal” for the District A‐small dataset, its solution does not visit one of the health centers.

**Table 5:** Computational comparison for the small datasets

	Optimal solution	MIP Gurobi^a^ (seconds)	MIP CBC^b^ (seconds)	MIP GLPK^b^ (seconds)	RoOT^b^ (seconds)
District A‐small	5.90	3.48	13.23	4.40^c^	1.02
District B‐small	6.69	4.31	100.61	60.00	1.88
District C‐small	6.20	12.35	13.23	540.20	0.90

a Time for optimal solution.

b Time to first discover the optimal solution.

c GLPK gave a different solution in comparison to the other three solvers: 4.63. However, its solution is infeasible, and does not visit one of the health centers.

**Fig. 12:**
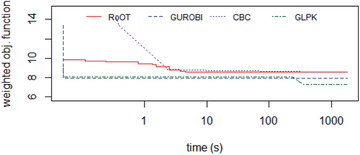
Solution comparison: District A (GLPK gave an infeasible solution smaller weighted objective function than the optimal calculated by Gurobi).

**Fig. 13:**
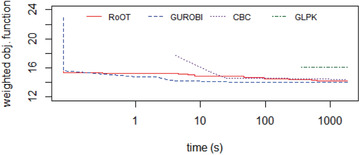
Solution comparison: District B.

**Fig. 14:**
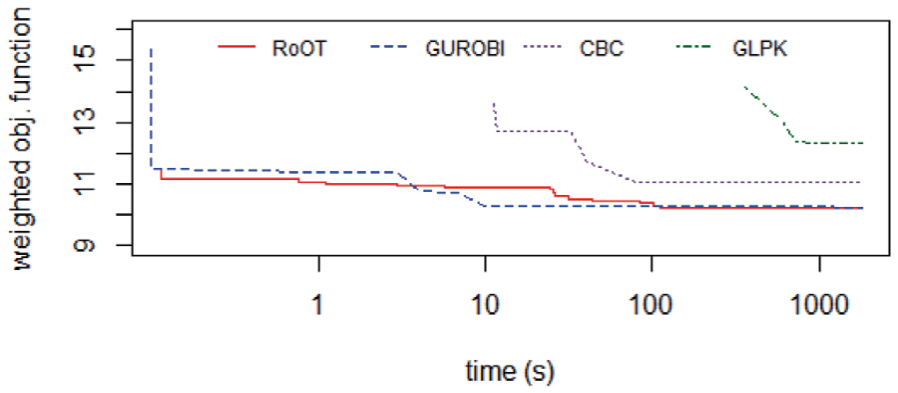
Solution comparison: District C.

The realistic datasets (District A, District B, and District C) were tested running the solvers for 30 minutes (i.e., 1800 seconds). Figures 12, 13, 14 provide plots of solution versus runtime (in seconds) for District A, District B, and District C, respectively. Note that the runtime is plotted in logarithmic scale. An end user typically expects a near‐optimal solution in less than two minutes (i.e., 120 seconds). Table 6 summarizes the best solution found in 30 minutes, with its lower bound, to provide the optimality gap.

**Table 6:** Computational comparison for five datasets

	District A		District B		District C		50 centers simple		50 centers modified	
	Best solution	Lower bound	Best solution	Lower bound	Best solution	Lower bound	Best solution	Lower bound	Best solution	Lower bound
RoOT	8.57	6.16	14.24	6.11	10.23	2.93	40.46	21.36	42.73	16.03
Gurobi	7.93	7.93	14.06	10.11	10.24	8.03	40.24	24.26	44.81	21.05
CBC	8.57	4.69	14.40	2.47	11.05	1.29	–	–	–	–
GLPK	7.30^a^	4.81	16.13	7.93	12.34	1.07	–	–	–	–

a GLPK gave a solution smaller than the optimal calculated by Gurobi, however, its solution is infeasible.

Gurobi found the optimal solution for District A, and RoOT had a performance similar to the CBC solver for that instance. For dataset District B, Gurobi, CBC, and RoOT had similar performances running for 30 minutes. For dataset District C, containing six vehicles, RoOT had better performance than all solvers, with small improvement over Gurobi.

However, as discussed in Section 3.4, VRPs are difficult to solve for large instances. To test the performance of RoOT in this situation, we created two test datasets with 50 centers and 5 vehicles. The distances are from the instance belgium‐road‐km‐d2‐n50‐k10 (Smet, 2017).

**Fig. 15:**
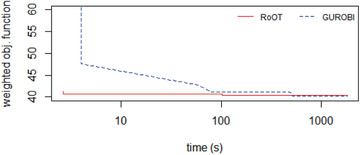
Solution comparison: 50 centers simple.

The first dataset had five identical vehicles with the same penalty values, and all the roads also had the same penalty values. Figure 15 shows solver performances. CBC and GLPK could not find a feasible solution in 30 minutes. RoOT found a feasible solution earlier than Gurobi, and Gurobi only reached the RoOT solution after 8.33 minutes (i.e., 500 seconds). However, Gurobi improved RoOT's solution and had a better solution at the end of 30 minutes. The difference in Gurobi's solution and RoOT's solution at 30 minutes was not significant (Fig. 15).

**Fig. 16:**
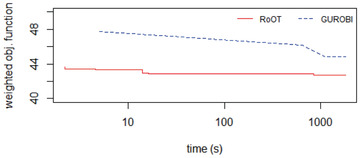
Solution comparison: 50 centers modified.

The second dataset had five different vehicles, with different capacities and penalties. Moreover, the penalties for the roads were also different. Again CBC and GLPK could not find a solution in 30 minutes. RoOT found a feasible solution and converged earlier than Gurobi, and by the end of the 30 minutes, Gurobi had not reached the RoOT solution (Fig. 16).

## 8 Conclusion

The main goal of this project is to create an easy‐to‐use routing tool that meets the needs of the users. The interactions between team members from the UW, VillageReach, and the Mozambican MoH were essential to reach this objective. Through many discussions and meetings, we developed the tool presented in this paper. RoOT is available on GitHub in English and Portuguese (Petroianu, 2019a, 2019b) as an open source for all users, especially those from NGOs, government, and academia. MoH users were trained to use the tool in January and February 2020, and the feedback received was favorable. However, full deployment has been interrupted due to the coronavirus pandemic.

RoOT gives good solutions in a timely manner. The final users do not have time or resources available to run an optimization model for hours or days to find the optimal solution. They want a good solution in one or two minutes, and RoOT is capable of that, as shown in this paper. Moreover, RoOT obtained good solutions within two minutes on the 50 center datasets. Scalability and speed are important factors for the users.

RoOT can be used for analysis, to evaluate changes in the situation (e.g., new vehicles or centers). RoOT can also be used operationally, or in emergencies, and in pandemics, such as COVID‐19, and, in addition, it can distribute other medical supplies, not only vaccines.

## 9 Future work

Considerations for future route optimization versions of RoOT include multiple day routes, with mixed transportation modes (e.g., land vehicles and boats) and island deliveries. This will require discussion on how intermediary storage of vaccines may be handled over multiple days. There is a risk of breakage and temperature range violation when unpacking and repacking vaccines in mid‐route for intermediary refrigeration. Mixed modes also present issues of coordination of timing as well as capacity issues.

One of the main challenges in preparing the data for the tool is to define the distance matrix. There is an opportunity to develop another tool that uses information from mapping and map APIs to populate the matrix, using names of locations, postal codes, or geographic coordinates.

The computational experience with RoOT has provided insights and ideas for future improvements. We will continue improving the indexing algorithm used in RoOT, to find better solutions faster and reducing the optimality gap with a tighter lower bound. We intend to test RoOT to evaluate its performance on more complex datasets with hundreds of centers.
